# Evaluation of locator versus TITACH attachments for mandibular 2-implant overdentures: a one-year randomized controlled clinical trial

**DOI:** 10.1590/1807-3107bor-2026.vol40.005

**Published:** 2026-03-06

**Authors:** Heba Wageh ABOZAED, Mohammed Ahmed ELADROSI, Moustafa Abdou ELSYAD

**Affiliations:** (a) Prince Sattam bin Abdulaziz University, College of Dentistry, Department of Prosthodontics, Riyadh, Saudi Arabia.; (b) Mansoura University, Faculty of Dentistry, Eldakahlia, Egypt.; (c) Mansoura University, Faculty of Dentistry, Department of Prosthodontics, Eldakahlia, Egypt.

**Keywords:** Bite Force. TITACH, Locator, Retention, Bite force, marginal bone loss, implant overdentures

## Abstract

Implant overdenture attachments serve as the crucial link between dental implants and removable dentures, significantly enhancing the stability, retention, and function of the prosthesis. This study was designed to assess the effectiveness of locator and TITACH attachments in retaining mandibular two-implant overdentures, maximum bite force, and marginal bone loss. The study involved 36 edentulous patients, each receiving new complete dentures (CDs) designed with the bilateral balanced occlusal concept. Two implants were positioned in the mandibular canine regions. Participants were randomly assigned to two equal groups: Group I (LOA) with overdentures using locator attachments, and Group II (TIA) with overdentures using TITACH attachments. Retention and maximum bite forces were measured using a digital force meter and bite force transducers at baseline (T0), and at 6 months (T6), and 12 months (T12) post-insertion. After insertion, marginal bone loss (MBL) was evaluated using digital periapical radiographs at T6 and T12. Retention significantly decreased over time across all groups (p = 0.024 for CD, p < 0.001 for LOA and TIA). Maximum bite forces significantly declined for LOA (p = 0.007) and TIA (p = 0.003) at all measurement intervals. TIA demonstrated the highest retention and bite forces, followed by LOA, while CDs exhibited the lowest retention and bite forces. TIA was associated with significantly greater MBL than LOA (p = 0.002 and p < 0.001 for TIA). TITACH attachments showed superior retention and maximum bite force compared to locator attachments. Both attachment types offered improved retention and maximum bite forces relative to complete dentures. Additionally, locator attachments were associated with less peri-implant bone loss than TITACH attachments.

## Introduction

Edentulism is a significant disability affecting older adults.^1^ Mandibular overdentures supported by two implants offer numerous benefits over traditional dentures, including improved retention and stability, greater chewing efficiency, preservation of the remaining alveolar ridges, increased patient comfort, satisfaction, and overall quality of life.^2^ Compared to fixed implant-supported prostheses, these overdentures provide more advantages at a reduced cost. Consequently, two-implant overdentures have been considered the minimal standard of care for edentulous patients.^3^

There are several commercial options for connecting implants to overdentures, including splinting devices (bars) and individual attachment systems such as balls, magnets, and resilient studs.^4^ The choice of attachment is influenced by various factors, including the required level of retention, available inter-arch space, patient’s manual dexterity, dentist’s expertise, and cost.^5^

Locator (resilient stud) attachments have self-aligning capability, a dual retention feature, and minimal inter-arch space requirements.^6^ Furthermore, the color-coded replaceable nylon inserts offer various retention levels. However, the nylon inserts are prone to significant wear and deformation, necessitating careful maintenance. In a recent 5-year randomized controlled trial by Elsyad et al., it was found that locator attachments had the highest rate of prosthetic complications, including wear, distortion, and the need for replacement of retentive components.^7^

The TITACH attachment (Implanova Dental Implants, Dental Evolutions Inc., Beverly Hills, USA) features metal-to-metal contact between the abutment and its cap. This system includes a silicone sleeve, a TITACH metal cap, and an abutment. The metal cap is designed with vertical openings that allow it to expand upon engaging with the abutment. During the cap’s pick-up process, the silicone sleeve serves as a block-out. This connection can accommodate up to 33 degrees of divergence for a single implant, or 66 degrees for implants positioned on opposite sides. Installing the cap requires a diameter of 6 mm and vertical clearance of 4.5 mm. It allows vertical cushioning up to 0.2 mm, facilitating gradual prosthesis seating and mucosa compression during use. Additionally, each attachment can withstand forces ranging from 7 to 10 pounds.^8^

The retention of the prosthesis is a crucial factor in the success of implant overdenture therapy.^9^ In a previous *in vitro* study,^10^ the authors discovered that TITACH attachments produced superior retentive force values compared to Locator attachments. The maximum bite force is influenced by the activity of the jaw’s elevator muscles, governed by cranio-mandibular biomechanics. For patients experiencing ridge resorption, dental implants can enhance masticatory efficiency by boosting bite force.^11^

Marginal bone loss (MBL) is a complex phenomenon that takes place around the cervical area of dental implants. Keeping track of MBL is crucial for evaluating the success of dental implants, as it is considered a dependable indicator of how bone responds to surgical procedures and occlusal forces. MBL plays a significant role in the onset of peri-implantitis, regardless of the underlying cause.^12^

We sought to evaluate and compare the effectiveness of locator attachments versus TITACH attachments in retaining mandibular implant overdentures, in addition to maximum bite force and marginal bone loss at different time intervals. The null hypothesis proposed that there would be no significant difference between the attachments under investigation.

## Methods

### Participant selection

**Figure 4:**
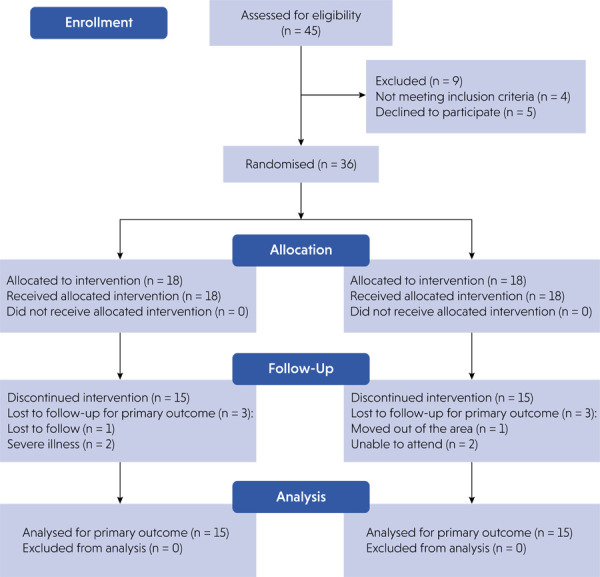
The study flow chart of participants.

The study involved 36 edentulous patients (20 males and 16 females) recruited from the outpatient clinic of the prosthodontics department at the Faculty of Dentistry, Mansoura University. Power analysis was conducted based on the findings of prior research.^13^ The authors identified a significant difference of 1 mm (SD = 0.53 mm) in marginal bone loss (in N) between the two implant overdenture attachments (power = 0.80, α = 0.05). The sample size was increased by 20% to account for potential dropouts. The present study was conducted following the CONSORT statement. The patient flow diagram and allocation for this study are reported in Figure 4.

The inclusion criteria were as follows: all patients had Angle’s class I maxilla–mandibular relation with sufficient inter-arch space (class II–IV according to Ahuja and Cagna)^14^, sufficient residual bone volume (class IV–VI Cawood and Howell)^15^ and quality (classes I–III, Lekholm and Zarb)^16^in the interforaminal area of the mandible (as verified by preoperative cone beam CT), patients complained of inadequate retention and stability of their conventional mandibular dentures. Exclusion criteria included systemic diseases hindering osseointegration, risky habits such as drinking or smoking, and a history of impaired neuromuscular control or recurrent TMJ problems.

The Ethics Committee at the Faculty of Dentistry at Mansoura University approved this clinical study with decision number (M10071020). This research was registered with ClinicalTrials.gov, Identifier: NCT06228859. All patients were informed about the detailed treatment plan and necessary follow-up appointments before signing the written consent forms. They received new maxillary and mandibular complete dentures designed with the bilateral balanced occlusal concept. To enhance neuromuscular adaptation, patients were instructed to wear these new dentures for six months before the overdenture insertion. The mandibular denture was duplicated using a transparent auto-polymerized acrylic resin to serve as a surgical guide.

### Surgical and prosthetic procedures

Following a standardized two-stage surgical procedure, two dental implants (Implanova Dental Implants, Dental Evolutions Inc.; Beverly Hills, USA; 13 mm in length and 3.7 mm in diameter) were placed bilaterally in the mandibular canine region. If a thin alveolar crest was encountered, the alveolar ridge was leveled to ensure sufficient bone width around the implants, and the site was closed with interrupted sutures (Vicryl, 0000). The mandibular denture was adjusted at the implant sites and relined with tissue conditioning material (Viscogel, Dentsply Sirona, York, USA), with the occlusion being refined. After a 3-month osseointegration period, healing abutments were placed to facilitate proper growth of the gingival tissues. The healing abutments were removed two weeks later, and the dentures were relined with hard acrylic resin. Participants were randomly divided into two groups (n = 18 per group) by blinded dental staff. Group I (LOA, n = 18) received overdentures with Locator attachments, while Group II (TIA, n = 18) received overdentures with TITACH attachments.

**Figure 1:**
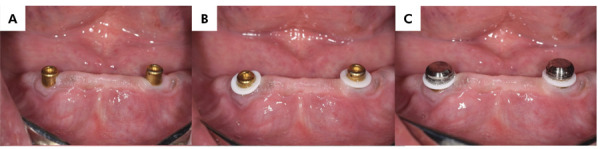
Locator overdentures. (a) Locator abutments in the patient’s mouth. (b) White blocking rings in position. (c) Metal housing in place.

**Figure 2:**
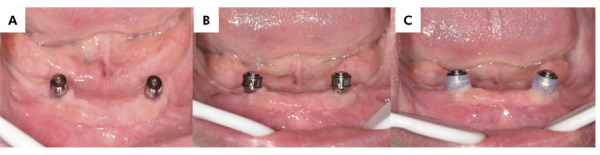
TITACH overdentures. (a) TITACH abutments in the patient’s mouth. (b) Metal housings over the TITACH abutments before trimming. (c) Cap-sleeve assembly in place.

For the LOA group (Figure 1), Locator abutments (Zest Anchors Inc., Carlsbad, CA, USA) were attached to each implant using a 25-Ncm torque. A white block-out spacer ring was then placed around each abutment, followed by the Locator metal housings fitted with black processing male components. For the TIA group (Figure 2), silicone ring sleeves were used to prevent the acrylic resin from locking around the TITACH abutment. These sleeves covered the entire neck of the abutment extending from the gingival area. The cap-sleeved assembly was securely snapped onto the TITACH abutment until fully seated. For both groups, the denture base was adjusted to avoid contact with the metal housings (LOA) or metal cap (TIA), and lingual vents were created in the denture to allow excess acrylic resin to escape during the pick-up process. The metal caps were secured to the dentures using auto-polymerized acrylic resin while the patients maintained centric occlusion with their maxillary and mandibular dentures. After the acrylic resin had set, the white blocking ring (LOA) or silicone sleeve (TIA) of the dentures was removed, and any excess acrylic resin was polished and finished. For the LOA group, the black processing male component was replaced with a transparent, medium retention insert,^10,17^ known as the locator nylon insert (2.270 g).

### Study outcomes

**Figure 3:**
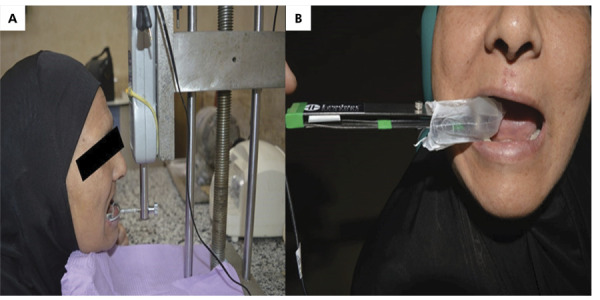
(a) Digital force-meter device for retention measurement. (b) Bite force transducer for masticatory bite force measurement.

Retention forces were measured utilizing a digital force meter affixed to a previously described apparatus, which exerted pure vertical dislodging forces on the mandibular overdenture apparatus.^18^ Self-curing acrylic resin was employed to attach four L-shaped hooks to the overdenture in the regions of the lower canine and first molar.^18^ Each participant, wearing mandibular dentures, was instructed to adjust their head position until the L-shaped hooks made simultaneous contact with the U-shaped fork while resting their chin on the equipment’s chin rest. Subsequently, the force meter was adjusted vertically until the overdenture was displaced from its original position (Figure 3a), at which point the force meter reading in newtons (N) was recorded. Following three repetitions of the measurements, the mean value was calculated.

Maximum bite forces were measured bilaterally utilizing a bite force transducer (iBite Digital Bite Force Sensor, Loadstar Sensors, Fremont, USA) (Figure 3b). The iBite force sensor represents an updated iteration of the widely used iBite force sensor, enabling clinicians or researchers to quantify the bite forces exerted by patients. Before use, the sensor was encased in a plastic sleeve, and metal surfaces and sensors were disinfected with an alcohol swab. The device employed is a bite fork equipped with strain gauges, positioned in the interocclusal space of the denture teeth in the first molar region. Measurements were conducted three times on each side of the patient, following instructions to bite down maximally on the device fork for a few seconds. The left and right force measurements were aggregated, and the mean was subjected to statistical analysis.

MBL was evaluated using digital periapical radiographs with the long cone parallel technique, employing a RINN XCP film holder (Rinn Co., Dentsply Sirona) under uniform settings for all patients. This methodology ensured the relative constancy of implant length across successive radiographs. The images, which were captured using a digital device (Digora, Soredex, Quakertown, PA, USA), were analyzed by a single examiner. MBL was determined as the distance from the implant platform level to the most coronal bone-to-implant contact visible in the digital radiograph. To measure MBL in millimeters (mm), the linear distance between the proximal crestal bone level and the implant shoulder was assessed at both the mesial (M) and distal (D) aspects of the implant. To account for magnification effects, the actual length of the implant (AL) was used to adjust the length of implants on a radiograph (RL). The most recent radiograph was compared with the previous one to detect marginal peri-implant bone loss. MBL was calculated using the proportional equation:^19^


$$MBL=\frac{M+D}{2}\times \frac{AL}{RL}$$

For CD, retention and maximum bite forces were evaluated at the time of insertion (T0) and at 6 months (T6) post-denture insertion. For LOA and TIA, retention forces, maximum bite force, and marginal bone loss were assessed at T0, T6, T12 post-overdenture insertion.

### Statistical analysis

Repeated measures ANOVA and paired samples t-test were employed to assess potential differences in retention, maximum bite forces, and marginal bone loss across observation times. Between-group comparisons were conducted using ANOVA and an independent t-test. P values were adjusted according to the Bonferroni method for multiple comparisons. Spearman’s correlation coefficient was utilized to determine the correlations of the retentive forces and maximum bite forces between groups. The overall threshold for significance (α) was set at 0.05. Data analysis was performed using SPSS® software version 25 (IBM SPSS Statistics for Windows, Armonk, USA).

## Results

No implant failures were observed, resulting in a 100% implant survival rate in both groups. Six patients were lost to follow-up. The analysis could not be completed for two patients in the LOA group due to severe illness, and another patient in the same group was lost to follow-up. In the TIA group, one patient relocated, and two patients were unable to attend the regular recall visits. Consequently, 15 patients in each group completed the analysis. The patient flow diagram is presented in Figure 4.

**Figure 5:**
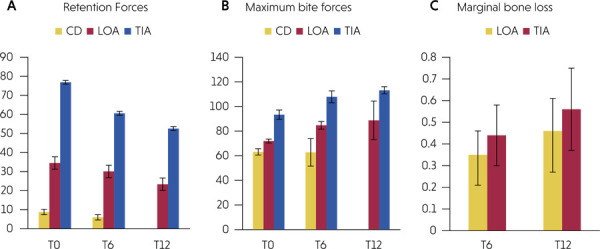
(a) Comparisons of retention forces between all groups. (b) Comparisons of maximum bite forces between all groups. (c) Comparisons of marginal bone loss between the LOA and TIA groups.

Table 1 and Figure 5a provide a comparative analysis of retentive forces across different groups and measurement intervals. A statistically significant variation in retentive forces was identified across all measurement intervals for each group. Specifically, for all groups, the highest retention forces were recorded at T0, followed by T6, with T12 demonstrating the lowest retention forces. A statistically significant difference was observed between each two measurement intervals for all groups, except the comparison between T0 and T6 within the CD group. A significant difference in retentive forces among the groups was evident at all measurement intervals (p < 0.001). Across all measurement intervals, the TIA group exhibited the highest retention forces, followed by the LOA group, with the CD group showing the lowest retention forces.

**Table 1:** Comparison of retentive forces (in N) between groups and measurement times.

Variable	T0		T6		T12		p-value
	X	SD	X	SD	X	SD	
CD	8.8**a**	1.30	6.0**b**	1.58	-	-	0.024^*^
LOA	34.54**a**	6.28	30.10**b**	3.30	23.36**c**	3.60	< 0.001^*^
TIA	76.88**a**	1.70	60.62**b**	2.69	52.60**c**	3.79	< 0.001^*^
p-value	< 0.001^*^		< 0.001^*^		< 0.001^*^		

X: mean, SD: standard deviation. CD: complete dentures, LOA: Locator overdentures, TIA: TITACH overdentures. T0: at the time of insertion, T6; 6 months after insertion, T12; 12 months after insertion. * *P* indicates significance at the 5% level. Different letters in the same row indicate a significant difference between measurement times (Bonferroni, p < 0.05)

Table 2 and Figure 5b present a comparison of maximum bite forces across different groups and measurement times. A significant variation in maximum bite forces was noted between measurement times for the LOA (p = 0.007) and TIA (p = 0.003) groups, while the CD group showed no significant difference in bite force between observations. In all groups, T0 recorded the highest maximum bite forces, followed by T6, with T12 showing the lowest. A significant difference in maximum biting forces among groups was evident at all measurement periods. Across all measurements, the TIA group had the highest maximum bite forces, followed by the LOA group, with the CD group showing the lowest forces.

**Table 2:** Comparison of maximum bite forces (in N) between groups and measurement times.

Variable	T0		T6		T12		p-value
	X	SD	X	SD	X	SD	
CD	63.20**a**	2.59	62.80**a**	11.23	-	-	0.708
LOA	72.00**a**	1.58	84.80**b**	3.11	88.80**b**	15.66	0.007^*^
TIA	93.40**a**	3.78	107.90**b**	4.83	113.20**b**	2.95	0.003^*^
p-value	< 0.001^*^		< 0.001^*^		< 0.001*		

X: mean, SD: standard deviation; CD: complete denture; LOA: Locator overdenture; TIA: TITACH overdenture. T0: at the time of insertion; T6: 6 months after insertion; T12: 12 months after insertion. * *P* indicates significance at the 5% level. Different letters in the same row indicate a significant difference between measurement times (Bonferroni, p < 0.05)

As shown in Table 3 and Figure 5c, marginal bone loss increased significantly over time (from T6 to T12) in both groups. The TIA group showed significantly higher marginal bone loss than the LOA group at T6 and T12. There was a non-statistically significant direct correlation between retentive forces and maximum bite forces across the groups as presented in Table 4.

**Table 3:** Comparison of marginal bone loss (in mm) between groups and measurement times.

Variables	T6		T12		p-value
	X	SD	X	SD	
LOA	0.35	0.11	0.44	0.15	0.002*
TIA	0.46	0.14	0.56	0.19	<0.001*
p-value	0.004*		0.009*		

X: mean, SD: standard deviation; LOA: Locator overdentures TIA: TITACH overdenture. T6; 6 months after insertion, T12; 12 months after insertion. *P indicates significance at the 5% level.

**Table 4:** Correlation between retentive forces and maximum bite forces between groups.

Variables	Correlation coefficient	p-value
CD		
T0	0.40764	0.09312
T6	0.22819	0.36245
LOA		
T0	-0.13214	0.63874
T6	-0.09286	0.74205
T12	-0.42143	0.1177
TIA		
T0	-0.31429	0.25394.
T6	-0.34643	0.2059
T12	-0.11786	0.67571

CD: complete denture; LOA: Locator overdenture; TIA: TITACH overdenture. T0: at the time of insertion; T6: 6 months after insertion; T12: 12 months after insertion.

## Discussion

The intraoral retention force measurement device addresses the limitations of conventional methods by providing pure vertical dislodging forces perpendicular to the occlusal plane and by preventing or tipping the overdentures during the measurement process. Furthermore, it facilitates the standardization of load application points.^18^

Overall, the null hypothesis was rejected in the current study. The retention forces of both attachment types exhibited a gradual decrease. This outcome was anticipated, as the loss of attachment retention following usage is a common problem associated with implant overdentures.^20^ Numerous studies have similarly documented a reduction in the retention forces of locator attachments over time.^21^ The metal-on-metal contact, which facilitates increased friction and wear, may account for the gradual decrease in retention observed in the TIA group. This reduction aligns with previous research on various metal attachment techniques.^22^ However, it is noteworthy that the rate of retention loss was more pronounced in attachment types incorporating plastic or nylon components compared to those constructed entirely of metal.^23^

The LOA and TIA groups demonstrated significantly higher retention rates than the CD group. This finding is consistent with other studies that have employed attachment techniques to enhance the stability and retention of overdentures supported by implants or teeth in edentulous or partially edentulous arches. Mandibular overdentures are highly recommended for LOA to optimize retention effects.^24^

In the current investigation, TIA was associated with greater retention forces than LOA. These findings corroborate those of a recent *in vitro* study^10^, which reported that the TITACH connection exhibited higher initial and ultimate retentive forces than the Zest Anchor locator attachment. Additionally, an in vivo study comparing direct and indirect TITACH attachment incorporation revealed that the TITACH attachment demonstrated favorable retention with both incorporation techniques, suggesting it as a suitable option for increased retention.^25^ This may be attributed to the robust nylon interface of the locator attachments, which generates lower friction and retention pressures compared to the metal-to-metal contact between the TITACH attachment components. Although metal-on-metal friction accelerates the wear of attachment components relative to resilient attachments,^26^ after six months of denture wear, the ultimate retention values of TITACH attachments remain superior to those of locator attachments. This observation is consistent with the findings of Ramadan and Mohamed^10^, who reported that following wear simulation, TITACH attachments exhibited greater ultimate retentive force values than locator attachments. This may be related to the design of the TITACH attachments, wherein the metal cap contains multiple metallic lamellae that engage with the circumferential undercut of the TITACH abutments. The stiffness of these lamellae appears to maintain retention forces even after wear, thereby reducing the need for metal cap reactivation. Conversely, locator nylon inserts are more susceptible to surface alterations and deterioration during operation, rendering them less resilient to wear and friction.^27^ Consequently, compared to other attachments, locator attachments are associated with a higher incidence of prosthetic complications, such as wear, distortion, and replacement of retentive components.^7^ Although TITACH attachments continue to exhibit higher retention values than locators after six months of use, the enhanced retention may transmit excessive stresses to the implants during displacement,^28^ potentially increasing marginal bone loss.

Maximum biting forces for both the LOA and TIA groups decreased over time. This decline may be related to the wear and deterioration of the nylon and metal caps used in the locator and TITACH attachments, which reduces their retention strength. It is well established that greater retention enhances biting force and masticatory efficiency.^29^

The LOA and TIA groups showed significantly higher maximum biting forces than the CD group. These findings are consistent with the York Consensus^3^, which indicated that implant mandibular overdentures offer superior retention and stability compared to conventional dentures. This increased retention improves chewing efficiency and boosts maximum bite forces.^30^The higher bite forces seen in the LOA and TIA groups can likely be attributed to the use of attachments that enhance overdenture retention and stability, along with better muscle function and food comminution.^31,32^ Research has demonstrated that patients with implant mandibular overdentures have substantially greater bite forces than those with traditional dentures.^33^ After implant placement, bite force doubles shortly after implant insertion and can increase by as much as 123% within one year.^32^Conventional mandibular dentures often lack stability, causing pain during chewing from pressure on the mucosa. This discomfort reduces muscle activity and weakens biting strength.^34^

TITACH attachments provide greater retention than locator attachments, allowing them to withstand higher biting forces while enhancing muscle thickness and activity.^35^Their metal-on-metal contact also improves the stability of the anterior denture. This increased retention and metal interface likely promote better axial transfer of chewing forces and generate significant occlusal forces before activating periosteal mechanoreceptors near the dental implant, which may increase maximum bite force.^36^ In contrast, the resilient locator insert supports the overdenture through the mucosa, causing most occlusal stresses to be absorbed by the mucosa. Additionally, wear of the nylon insert reduces retention, potentially leading to more mucosal irritation and lower maximum bite forces.^37^

The results of this study suggest that there may be a statistically significant difference between groups I and II after 6 and 12 months of implant loading with a mandibular overdenture. It appears that there may be an increase in bone resorption in the TITACH group compared to the locator group. It seems that the difference in the matrix-patrix connection of the two attachments around which the denture rotated may have resulted in less crystal bone resorption around the implant supporting the locator attachment compared to the TITACH attachment.^38^

It is likely that the locator attachment group was able to reduce crystal bone resorption because of the supra-radicular design of the locator, which moved the fulcrum point closer to the fixture and lowered the lever arm and torque.^39^ Because of its design, which allows for 0.2 mm of vertical resilience and 8 degrees of hinging in either direction, locator attachments have the advantage of being able to move in both the vertical plane and hinge axis. It is therefore possible to distribute pressures evenly along the long axis of the implant with this locator.^40^ Another factor that may contribute to the increased retention in the TITACH attachment group is that numerous researchers have reported that the retentive forces of an attachment system influence the stresses generated in the peri-implant bone during loading. It has been observed that as retention increases, greater loading forces are applied to the implant/abutment complex.^41^

The limitations of the current study involve the short evaluation periods. A longer follow-up would provide better insights into the effectiveness of attachments used to support mandibular overdentures. A more comprehensive follow-up study would provide valuable insights into the effectiveness of the attachments used to support mandibular overdentures. Future clinical research will be instrumental in evaluating peri-implant health and the maintenance of prosthodontics for mandibular overdentures supported by two implants with varying attachment types. Overall, the null hypothesis was rejected in the current study.

### Clinical implications

Implant overdentures retained either with locator or TITACH attachments may serve as an alternative treatment for patients seeking improved retention and increased bite forces. From a biomechanical perspective, the design of the prosthesis enhances retention and bite force while redistributing stress to the dental implants. This highlights the necessity for enhanced monitoring to prevent subsequent peri-implant bone loss.

## Conclusion

TITACH attachments showed superior retention and maximum bite force compared to locator attachments. Both attachment types offered improved retention and maximum bite forces relative to complete dentures. Additionally, locator attachments were associated with less peri-implant bone loss than TITACH attachments.

## Data Availability

The authors declare that all data generated or analyzed during this study are included in this published article.
